# Sporadic Creutzfeldt-Jakob Disease among Physicians, Germany, 1993–2018

**DOI:** 10.3201/eid2608.191159

**Published:** 2020-08

**Authors:** Peter Hermann, Johannes Treig, Steffen Unkel, Stefan Goebel, Timothy Bunck, Martha Jünemann, Tim Friede, Inga Zerr

**Affiliations:** University Medical Center Göttingen, Göttingen, Germany (P. Hermann, J. Treig, S. Unkel, S. Goebel, T. Bunck, M. Jünemann, T. Friede, I. Zerr);; German Center for Neurodegenerative Diseases, Göttingen (I. Zerr)

**Keywords:** Creutzfeldt-Jakob disease, prion diseases, epidemiology, risk factors, healthcare professions, Germany, physicians, prions and related diseases

## Abstract

We investigated sporadic Creutzfeldt-Jakob disease (sCJD) among physicians in Germany by analyzing occupational information of patients with sCJD recorded by the German CJD Surveillance Unit (1993–2005; 1,250 patients, of whom 4 [0.32%] were physicians) and the National Reference Center for Human Spongiform Encephalopathies (2006–2016; 1,491 patients, of whom 13 [0.87%] were physicians). Among the physicians, we did not identify any neurologists, neurosurgeons, psychiatrists, or pathologists. A cumulative sum test showed an increase in reported physicians over time. Data for 2017–2018 indicated an increased rate of physicians among all notified sCJD cases (5/239 [2.1%]) when we used the total population of Germany as control group. Our data suggest the possibility of an increased risk for sCJD among physicians in Germany. However, we can only speculate about the reasons, and larger multinational studies are needed to replicate the finding and to clarify whether this finding is a general or a country-specific phenomenon.

## Abstract 2

sCJD patients were significantly more likely than the general population to be physicians.

Creutzfeldt-Jakob disease (CJD) is a syndrome comprising dementia and various neurologic signs and symptoms (*1*) caused by the transmissible misfolded prion protein scrapie (*2*). Reported death rates and incidence rates differ from 1.67 (*3*) to >2 per million person-years (*4*,*5*). In contrast to animal prion diseases (*6*,*7*), transmitted human prion diseases are uncommon. Variant CJD (vCJD) caused by ingestion of beef is rare (231 cases worldwide) (*8*), and its incidence has decreased since 2000 (*9*). Most cases of human prion disease are sporadic CJD (sCJD; 84%–93%), followed by genetic CJD (5%–10%). Only <4% are considered to be iatrogenic (iCJD) (*3*,*10*–*12*). Clinical diagnostic criteria of iCJD imply the presence of an iatrogenic risk factor (*13*). Known cases were caused by cadaver-derived growth hormones, dura mater grafts, neurosurgical instrument contamination, and corneal grafts (*12*). On the other hand, iCJD might be overlooked when no classic risk factor is present. Neuropathologic characteristics can identify iCJD only in a subgroup of cases (*14*,*15*). Unrecorded cases related to surgery are likely because an increased risk for sCJD in persons with a history of surgery was reported (*16*); however, data on this issue remain ambiguous (*17*). vCJD transmitted by transfusion of blood products has been reported (*8*), but no confirmed case was recorded among recipients of blood from donors with sCJD (*18*–*20*).

An increased risk for iCJD among caregivers and healthcare professionals has been suggested, but its evaluation is complex (*21*–*24*). Previous studies neither unequivocally displayed nor ruled out relevant increases in risk for CJD among healthcare professionals (*25*,*26*). Furthermore, these investigations were mostly designed as case–control studies, which are prone to bias because of case selection. Therefore, we aimed to evaluate sCJD among physicians using historical epidemiologic data from 25 years of CJD surveillance in Germany and the whole population of that country as controls.

## Methods

### Study Design and Data Acquisition

In the framework of a retrospective cohort study, we evaluated 4,645 patient files representing all suspected CJD cases reported to the German surveillance group during June 1993–December 2016 about the patient’s occupational history to identify physicians of all specialties. In addition and as negative controls, we collected information about other professions.

The centralized assessment of suspected human prion diseases in Germany started in June 1993 and was conducted by the CJD Surveillance Unit of the University Medical Center Goettingen. Since January 2006, health authorities have officially charged this center with CJD surveillance and named it the National Reference Center for Human Transmissible Spongiform Encephalopathies (NRZ-TSE). In Germany, notification of sCJD is required. Health authorities advise clinical institutions to contact the NRZ-TSE for clinical classifications of notified cases. The NRZ-TSE counsels physicians with respect to differential diagnosis and hygienic issues and records clinical data, including the patient’s professional background. Specifically, until 2006, physicians from the CJD Surveillance Unit visited and interviewed patients and caregivers using a standardized questionnaire.

### Patient Cohorts

We considered all patients in the database for this study. Inclusion criteria for further analyses were diagnosis of probable or definite sCJD according to World Health Organization criteria (*2*) and age >35 years. We reviewed all available questionnaires (evaluated by the NRZ-TSE) and medical reports (sent to the NRZ-TSE by treating institution) since 1993 for patient’s professions to identify physicians. During 1993–2005, the research group of the University Medical Center Goettingen had to actively search for suspected CJD cases (e.g., through regular newsletters to all neurologic and psychiatric centers in Germany). Most reported patients had been visited by physicians from the research group, and epidemiologic questionnaires were available for analyses. In 2006, the group was assigned as National Reference Center, leading to a substantial increase in reported cases and resulted in a decrease in the proportion of visitations and interviews. Because of these structural differences, we divided the study cohort into cohort A (reported 1993–2005; 1,250 persons) and cohort B (reported 2006–2016; 1,491 persons) and analyzed them separately.

### Population-Based Cohorts

We used publicly available data on the population of Germany to create control groups in each time frame (matching cohorts A and B). Numbers of working and retired physicians were sourced from the database of the German Federal Medical Association (Bundesärztekammer [BÄK], Berlin, Germany), which provides the number of physicians and information about age, sex, specialty, and location. Membership is required for, but is not restricted to, all working physicians and does not expire with retirement. To analyze the entire population, we obtained numbers from the German Federal Office of Statistics.

### Data Analyses and Statistical Methods

BÄK data give numbers of physicians in Germany in different age categories. The youngest category was <35 years of age (without further differentiation). Only 10 patients in the sCJD cohort were <35 years of age, and none were physicians. We included only patients and controls >35 years of age to achieve approximate age matching between the German physicians and the sCJD cohort. We did not further stratify for age (and sex) because of the low case count among physicians with sCJD. We pooled all data from our sources using Excel 2016 (Microsoft, https://www.microsoft.com). We used Statistica (https://www.statsoft.de) for descriptive analyses and performed further statistical analyses using the statistical software R version 3.4.2 (https://www.r-project.org). We considered results with p<0.05 to be significant. 

The aims of the study were to evaluate the rate of physicians in the cohort of CJD patients and to investigate a potential risk modification using population-based data. The aims had been framed before data collection. We used Fisher exact test to compare the number of physicians in the cohort of CJD patients and the number of physicians in the population of Germany. 

Analyses were performed as follows. To define the number of nonphysicians in the CJD cohort, we considered only patients with known occupation. These analyses were based on the assumption of a corresponding number of physicians in the group of patients with unknown occupation. We further considered all CJD patients assuming that no additional physicians were in the group of patients with unknown occupation. We used the results to perform sensitivity analyses to evaluate the number of physicians in the group of patients with unknown occupation that would be necessary to reach statistical significance using Fisher exact test. We conducted a CUSUM (cumulative sum)–based test for a change point in a time series (*27*) to investigate alterations of the number of reported physicians with CJD over time (per year). Finally, in an additional step, we collected data from 2017 and 2018 and analyzed them to validate results of the previous analyses on the basis of the historical cohorts (1993–2016).

## Results

### Descriptive Data Analyses: CJD Cohort

**Figure 1:**
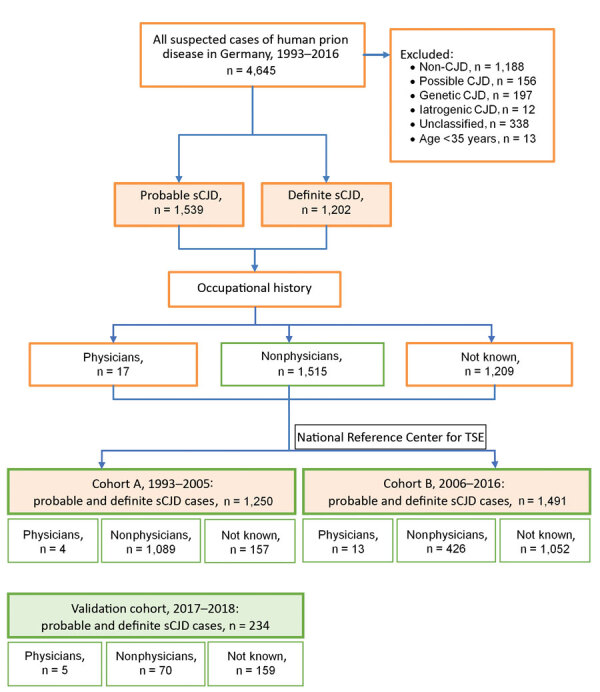
Study cohort and case selection in a study of sCJD among physicians, Germany, 1993–2018. All case numbers are based on the classifications of the German National Reference Center for Human Transmissible Spongiform Encephalopathies (*2*) in February 2019. CJD, Creutzfeldt-Jakob disease; sCJD, sporadic CJD; TSE, transmissible spongiform encephalopathy.

**Figure 2:**
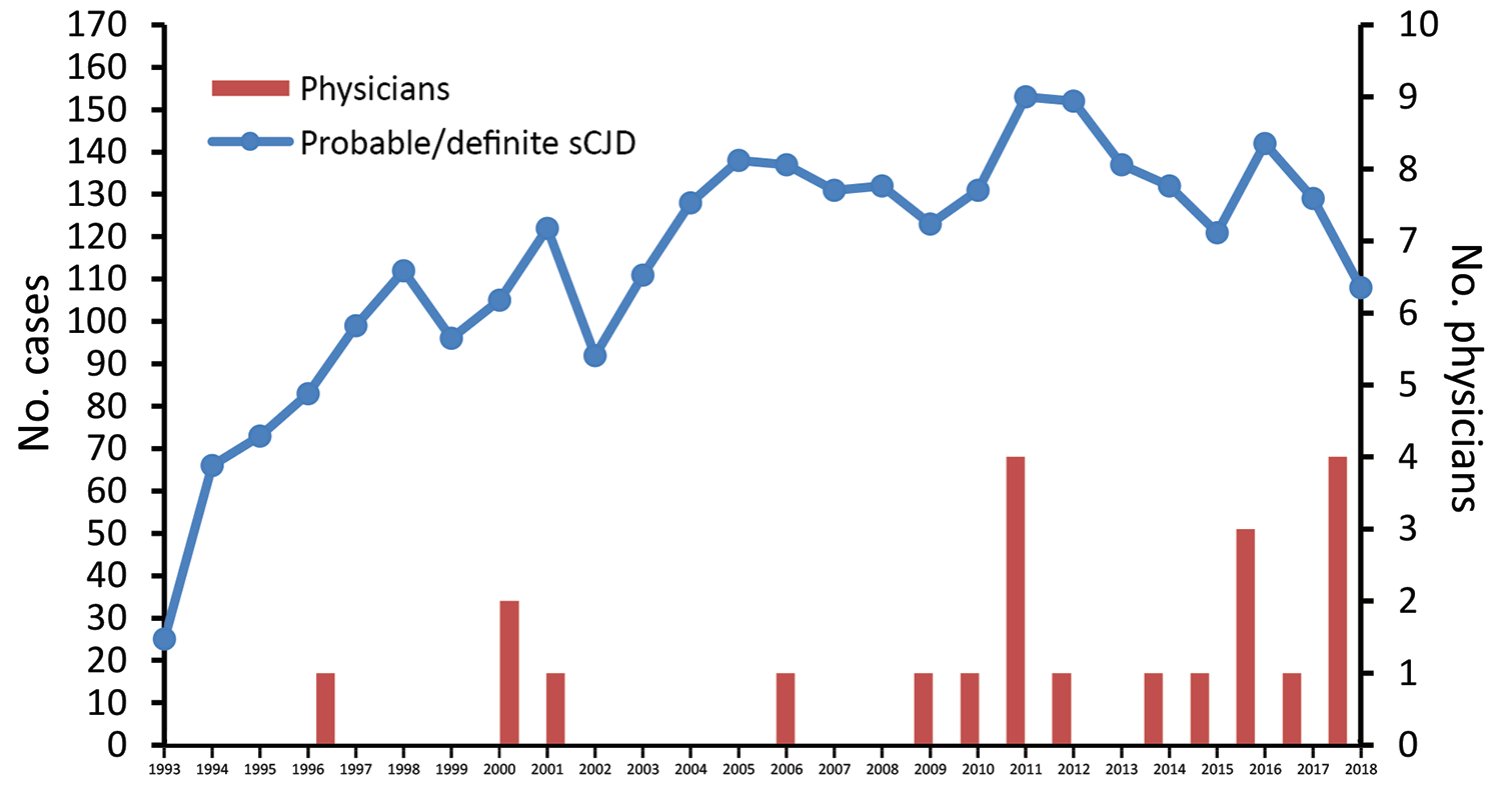
Definite and probable sCJD cases and number of physicians with sCJD, Germany, 1993–2018. All case numbers are based on the classifications of the German National Reference Center for Human Transmissible Spongiform Encephalopathies (*2*) in February 2019. Red bars, number of physicians reported in 1 year; blue line, number of probable and definite sCJD cases per year. sCJD, sporadic Creutzfeldt-Jakob disease.

Of 4,645 suspected CJD cases during June 1993–December 2016, we classified 2,754 as probable sCJD (1,543) or definite sCJD (1,211). We classified other cases as possible sCJD (*2*) (156 cases), non-CJD (1,188), genetic prion disease (197), and iCJD (12). A total of 338 reported cases remained unclassified because of incomplete clinical information. We reduced the number of probable and definite sCJD cases to 2,741 after excluding patients <35 years of age. We determined occupation for 1,532 (55.9%) patients, of whom 17 (1.1%) were physicians (Figures 1, 2).

In cohort A (June 1993–December 2005), we classified 539 (43%) of the 1,250 cases as probable sCJD and 711 (57%) as definite sCJD. In cohort B (January 2006–December 2016), we classified 1,000 (67%) of the 1,491 cases as probable sCJD and 491 (33%) as definite sCJD. The mean age of cohort A patients was 66 years (range 35–90 years) and of cohort B patients was 68 years (range 37–93 years). In cohort A, 58% of patients were women; in cohort B, 52%. Information about codon 129 polymorphism was available for 1,039 (83%) cohort A cases and 581 (39%) for cohort B cases (Table 1). For 1,532 (56% in cohorts A and B combined) patients, we were able to evaluate history of occupation before illness (cohort A, 1,093 patients; cohort B, 439). For some patients, >2 different professions were recorded (up to 7). We considered occupation as a physician at any point in time. Occupation as a physician was known for 4 cohort A patients (0.3% of all patients; 0.4% of cohort A patients) and 13 cohort B patients (0.9% of all patients; 3% of cohort B patients).

**Table 1:** Characteristics of sCJD patients, Germany, 1993–2018*

Variable	Cohort A	Cohort B	Validation cohort
Time period	1993 Jun–2005 Dec	2006 Jan–2016 Dec	2017 Jan–2018 Dec
Total, no. (%)	1,250 (100)	1,491 (100)	234 (100)
Definite sCJD	711 (57)	491 (33)	59 (25)
Probable sCJD	539 (43)	1,000 (67)	175 (75)
Mean age, y (range)	66 (35–90)	68 (37–93)	68 (41–91)
Sex			
F	731 (58)	769 (52)	118 (50)
M	519 (42)	722 (48)	116 (50)
Codon 129, no. (%)			
MM	693 (67)	322 (55)	8 (53)†
MV	180 (17)	134 (23)	7 (47)†
VV	166 (16)	125 (22)	0
Occupation known, no. (%)	1,093 (87)	439 (29)	70 (30)
Physicians, no. (% of all patients, % of known occupation)	4 (0.3, 0.4)	13 (0.9, 3)	5 (2.1, 7.1)

*MM, methionine homzygosity; MV, heterozygosity; sCJD, sporadic Creutzfeldt-Jakob disease; VV, valin homozygosity. †Since 2015, codon 129 analyses were not regularly performed during neuropathologic investigation.

Physicians with sCJD (including patients from the validation cohort 2017–2018) had a broad spectrum of medical specialties. Surgical specialties were present for 14 patients (surgery without information about further specialty, 3 patients; trauma/orthopedic surgery, 5; gynecology and otolangyrology, 2 each; urology and visceral surgery, 1 each). The others had nonsurgical specialties (internal medicine, 5; anesthesiology, podiatry, and general practice, 1 each). Of all physicians with sCJD (1993–2018), 64% had a surgical specialty (Table 2); in 2018, only 31% of all physicians in German had a surgical specialty (*28*). Very long duration of disease occurred only among physicians with surgical specialties (mean 205 days vs. 109 days for nonsurgical specialties; overall 175 days [range 49–809 days]). We identified no hospital in Germany that had employed >1 physician with sCJD, but a complete occupational history was not available for all patients, especially in cohort B. We found no link between a physician and another known sCJD patient, but only limited information was available (Appendix Table). Most patients were not able to give detailed information about this issue because of progressed cognitive impairment. The rate of autopsy-confirmed cases was 55%. Prion typing was performed in only 4 cases.

**Table 2:** Characteristics of physicians with sCJD, Germany, 1993–2018*

Variable	Total, N = 22	Surgical, n = 14	Nonsurgical, n = 8
Population-based %†	100	39	61
Sex, no. (%)			
F	3 (14)	2 (14)	1 (12)
M	19 (86)	12 (86)	7 (88)
Mean age, y (range)	67 (53-83)	65 (53-83)	69 (60–75)
Classification, no. (%)			
Definite	12 (55)	7 (50)	5 (63)
Probable	10 (45)	7 (50)	3 (38)
Mean duration of disease, d (range)§	175 (49-809)	205 (84-809)	109 (49–272)
Codon 129, no.			
MM	7	5	2
VV	2	2	0
MV	2	1	1
NA	11	6	5

*MM, methionine homzygosity; MV, heterozygosity; NA, genotype not available; sCJD, sporadic Creutzfeldt-Jakob disease; VV, valine homozygosity. †Percentage of surgical and nonsurgical specialties among all physicians in 2018 (*28*). §Information about disease duration (onset to death) was available for 21 patients,

### Descriptive Data Analyses: Population-Based Cohort

The number of physicians in Germany increased from 297,803 in 1993 to 496,240 in 2016 (*29*), a factor of 1.67. For each period corresponding to cohort A (1993–2005) and cohort B (2006–2016), mean values of yearly numbers were calculated that excluded physicians <35 years of age. Mean numbers were 295,556 (range 240,709–345,599) during 1993–2005 and 382,558 (range 349,878–416,311) during 2006–2016. We performed the same calculations considering the entire population of the same age in Germany (*30*): mean 47,907,927 (range 44,336,444–51,243,273) during 1993–2005 and 51,601,356 (range 51,553,192–51,961,175) during 2006–2016.

### Rate of Physicians in sCJD Cohorts and in the Total Population of Germany

We based contingency tables on the numbers of all patients in the study cohort for whom occupation was known (cohort A, 1,093 patients; cohort B, 439 patients), all physicians in the sCJD cohort (cohort A, 4 patients; cohort B, 13 patients), the population of Germany, and all physicians in that population (Table 3). Fisher exact test yielded an odds ratio (OR) of 0.59 (95% CI 0.16–1.52; p = 0.44) for cohort A and OR 4.09 (95% CI 2.16–7.06; p<0.001) for cohort B (Table 3). These results indicate a significantly higher rate of physicians in cohort B than in the total population of Germany.

**Table 3:** Physicians in cohorts of sCJD patients and in the whole population, Germany, 1993–2018*

Variable	Physicians†	Nonphysicians†	OR (95% CI)‡	p value
Cohort A, 1993–2005)				
sCJD patient				
Known occupation	4	1,089	0.59 (0.16–1.52)	0.44
All	4	1,246	0.52 (0.14–1.33)	0.27
German population	295,552	47,611,282		
Cohort B (2006–2016)				
sCJD patient				
Known occupation	13	426	4.09 (2.16–7.06)	<0.001
All	13	1,478	1.18 (0.63–2.02)	0.54
German population	382,545	51,218,372		
Validation cohort (2017-2018)				
All sCJD patients	5	234§	2.61 (1.08–6.33)	0.05
German population	424,413	51,924,527		

*OR, odds ratio; sCJD, sporadic Creutzfeldt-Jakob disease. †>35 years of age. ‡By Fisher exact test. §Information about occupation was available for 88 patients.

We based this approach on the assumption of a corresponding proportion of physicians and nonphysicians in the group of sCJD patients without known occupational history. In a second step, we included the entire study cohort, assuming there were no additional physicians in the group of sCJD patients for whom occupational history was not known. Cohort A did not differ significantly from the total population of Germany (OR 0.52 [95% CI 0.14–1.33]; p = 0.27); likewise, cohort B did not differ significantly from the total population of Germany (OR 1.18 [95% CI 0.63–2.02]; p = 0.54). Subsequently, we conducted a sensitivity analysis to determine the number of physicians in the group without known occupation who would be required for a statistically significant difference between the study cohort and the total German population: 9 for cohort A (p = 0.03) and 5 for cohort B (p = 0.047). In a forth step, we investigated the change of the rate of reported physicians in the study cohort over time: 0.32% for cohort A (1993–2005) and 0.87% for cohort B. Results of our CUSUM test showed an increase of reported cases (p = 0.04) and identified a change point from 2008 to 2009.

### Postanalytic Evaluation of 2017 and 2018

In 2017 and 2018, a total of 239 sCJD patients were reported (129 in 2017, 110 in 2018). We identified 5 physicians (1 in 2017, 4 in 2018) (Figure 2). Including the entire postanalytic cohort (sCJD patients 2017–2018), regardless of known occupational history and using population data from 2017 (29,30), excluding patients <35 years of age, we found a significantly elevated rate of physicians among sCJD patients (OR 2.61 [95% CI 1.08–6.34]; p = 0.05 by Fisher exact test) (Table 3).

## Discussion

Although prion diseases are transmissible, homozygosity for methionine at codon 129 (an intrinsic factor) is the only established risk factor for sCJD (*11*,*31*). Case–control studies have shown slightly elevated ORs for several features; for example, work at an animal laboratory, ophthalmologic surgery (*32*), ingestion of raw meat and brain (*24*), and history of brain surgery (*33*) (Table 4). Being employed as health professionals was a risk in a meta-analysis of case–control studies (*34*) but was not confirmed in a later prospective study (*23*). Because of the methodologic approaches used, most results were nonsignificant or prone to biases (*17*,*43*). Only 1 study used large population-based data from a US death registry (6 million cases screened, 636 CJD cases and 3,180 controls selected) and identified working as a butcher and work in physicians’ offices as occupational risk factors (*35*). Other investigations of occupational risk factors for sCJD are not available, but the presence of unpublished data that might show inconclusive or null results cannot be excluded. Data on the development of reported cases over time with respect to occupational history are not available.

**Table 4:** Review of studies about health professionals and CJD*

Study or report	Observation or sCJD patient
Case–control study	
Wientjens et al., 1996 (*34*)	Observation: 1975–1984 (Japan, United Kingdom, United States), meta-analysis of 3 case–control studies
	• 178 cases, 333 controls (hospital based, community based, and spouses)
	• Nonsignificantly increased risk for health professionals (OR 1.5 [95% CI 0.5–4.1])
Van Duijn et al., 1998 (*24*)	Observation: 1993–1995 (France, Germany, Italy, Netherlands, United Kingdom)
	• 405 cases, 405 controls (hospital-based)
	• No increased risk for health professionals (OR 0.92 [95% CI 0.69–1.32])
Cocco et al., 2003 (*35*)	Observation: 1984–1995 (United States)
	• 636 cases, 3,180 controls (population-based from a death registry)
	• Increased risk for workers in physicians’ offices (OR 4.6 [95% CI 1.2–17.6])
Ruegger et al., 2009 (*32*)	Observation: 2001–2004 (Switzerland)
	• 69 cases, 224 controls (from general practitioners and random digit telephone dialing)
	• Nonsignificantly increased risk for medical professionals (OR 1.46 [95% CI 0.43–5.15])
Case report	
Schoene et al., 1981 (*36*)	1 neurosurgeon
Miller, 1988 (*37*)	1 histopathologist
Sitwell, 1988 (*38*)	1 histopathologist
Gorman et al., 1992 (*39*)	1 pathologist
Berger et al., 1993 (*40*)	1 internist (with training in pathology 30 y before disease onset)
Weber et al., 1993 (*41*)	1 orthopedic surgeon (handling dura 20–24 y before onset)
Mitrova et al., 2000 (*42*)	1 physician, 5 nurses, 1 medical technician, 1 ambulance driver
Alcalde-Cabero et al., 2012 (*25*)	Observation: 1965–2010; case reports and literature review
	• 202 health professionals (among 8,321 cases)
	• 65 physicians (9 general practitioners, 7 surgeons, 7 internists, 4 dentists, 3 ophthalmologists, 2 pathologists), and 137 other health professionals

*OR, odds ratio; sCJD, sporadic Creutzfeldt-Jakob disease.

In addition to selection bias, the lack of studies that could validate occupational risk factors for sCJD might be caused by multiple comparisons of too many variables causing insignificant results. For this study, we focused on the evaluation of employment as physician as potential risk factor for sCJD. We used data from a prospective epidemiologic surveillance database in Germany and population-based data as controls.

Our first analysis showed a significantly elevated rate of physicians in the study cohort (OR 4.09; p<0.001) during 2006–2016 (cohort B), the years in which structured epidemiologic surveillance had been conducted. The exclusion of patients with unknown occupational history represents a case–control design limited by the possibility of selection bias. An occupation as physician may be more likely to be reported than others; on the other hand, only clinical data relevant for case classification were available for many patients from cohort B. The study design for cohort A was more precise because most suspected patients were examined in person in notifying hospitals, whereas for cohort B, only a proportion of sCJD patients (27%) were examined.

Nonetheless, our second analysis of the entire CJD population and found no significant results (OR 0.53 [p = 0.27] for cohort A; OR 1.18 [p = 0.543] for cohort B). Therefore, we performed a sensitivity analysis indicating 5 additional physicians in the group with unknown occupational history (1,052 [71%]) in cohort B who would be necessary for a significant result. This number was higher in cohort A (9 patients), although the number of patients in cohort A with unknown occupation was much smaller (157 [13%]). These findings suggest that the number of reported physicians with sCJD increased in later years, whereas the reported number of sCJD cases was stable. We validated this finding with a CUSUM test (p = 0.04, change point from 2008 to 2009). During 2017–2018, the increased rate of sCJD in physicians was significant, even when we included all 239 reported cases in the analysis (OR 2.61; p = 0.05).

Fourteen of the 22 physicians were surgeons, but none had worked in neurosurgery and only 1 had worked in a neuropathology department for 1 year. This finding is remarkable because the high proportion of surgeons (64%) versus nonsurgeons (36%) in the sCJD group differs from the population control (39% vs. 61%). The apparent differences of clinical characteristics (age of onset, disease duration) might be explained by slightly different distribution of codon 129 genotypes in the 2 groups. Because of the low number of cases, we could not investigate these observations further. In most cases, information about genotype, prion type, and neuropathologic characteristics was insufficient to identify or exclude iCJD. No neurologists or psychiatrists were reported. We could not find regional links within the group of physicians or with other sCJD patients who had received surgical interventions and might have been index patients for obscure iCJD. Thus, we were not able to establish a causal relation between the statistical risk factor (occupation as a physician) and the disease. Nonetheless, this finding must be interpreted with regard to potential incubation times of up to 30 years (*10*) and incomplete information about residence history in most patients from cohort B.

Although the use of a very large cohort of patients with sCJD and a population-based control group is a strength of our investigation, the study has several limitations. Because of the low number of physicians with sCJD, every bias in the case group would cause an immense effect on statistical analyses (e.g., unrecorded cases, misdiagnosed cases). Thus, we must interpret our results cautiously. Definite (neuropathologic) diagnosis was available only for some cases in our study, but the high accuracy of clinical diagnoses performed by our center has been reported previously (*5*). The altered status of the surveillance group after it was named a National Reference Center in 2006 might be a source of bias. We cannot exclude that the surveillance system in Germany has improved over the years, but the available data of patients’ occupations has decreased in recent years (Table 1), which makes an underestimation of the number of physicians before 2006 highly unlikely. An increased awareness for CJD among German physicians resulting in more reported cases in recent years is also unlikely regarding the decreasing worldwide incidence of vCJD since 2000.

Another limitation of our study is the lack of further and more detailed statistical analyses. We could not calculate individual ORs for certain medical specialties. Only an extremely large-scale study pooling data from multiple national reference centers would be capable of doing that. In addition, we were not able to stratify ORs by age and sex. The age cutoff of >35 years was an attempt only to achieve an approximate matching of age in the case and the control groups. On the other hand, recorded physicians with sCJD showed a strong tendency to be male and have an age at onset of 60–75 years. In this context, unstratified analyses might underestimate ORs. Another limitation of the study is that we analyzed only 1 occupational risk factor. Other professionals, such as laboratory scientists or nurses, should be carefully considered, but the lack of data (especially population-based figures) prevented from further analyses.

The high proportion of physicians among patients with sCJD and its increase over the last years were displayed in a statistical model based on data from the population of Germany. We showed that sCJD patients were significantly more likely than the general population to be physicians, suggesting that it might be an occupational risk factor. Previous epidemiologic studies have not clearly identified an elevated risk for sCJD among physicians (Table 4), but the most recent available data are from 2010. Our study yielded significant results only after 2005, and the CUSUM test identified an increased number of physicians with sCJD after 2008. No specialties involved specifically in treating patients with CJD have been reported. Nonetheless, we found that a high proportion of physicians with sCJD were surgeons, although we can only speculate about the reasons. A larger study comprising new data from other countries is needed to clarify whether this finding is a general or a country-specific phenomenon.

Appendix All physicians reported to have sporadic Creutzfeldt-Jakob disease, 1993–2018.
