# Supplementary material for: Sporadic Creutzfeldt-Jakob Disease among Physicians, Germany, 1993–2018
Source: Emerg Infect Dis. 2020 Aug;26(8):1710–9. doi: 10.3201/eid2608.191159 (PMC7392457; doi:10.3201/eid2608.191159)
Supplement: Appendix — All physicians reported to have sporadic Creutzfeldt-Jakob disease, 1993–2018. [file 19-1159-Techapp-s1.pdf]

Article DOI: https://doi.org/10.3201/eid2608.191159

Sporadic Creutzfeldt-Jakob Disease among Physicians, Germany, 1993–2018

Appendix

Appendix Table. All physicians with sporadic CJD (1993–2018)*

Age, y/sex	CJD diagnosis	Codon 129	Prion type	Duration, d	CSF 14–3-3	MRI†	EEG†	CSF RT-QuIC	Neuropathologic findings	Specialty
63/M	Definite	MV		272	+	+	–	NA	Deposition of PrP (biopsy)	Internal medicine
55/M	Definite	MM		84	+	NA	+	NA	Deposition of PrP, no unusual pattern	Abdominal surgery, neuropathology
73/M	Probable			377	+	+	+	NA		Urology
60/M	Definite			61	+	+	+	NA	Deposition of PrP, no unusual pattern	Internal medicine
71/M	Probable	MM		110	+	–	+	NA		Anesthesiology
56/M	Definite			86	+	+	–	NA	Morphological/histochemical pattern as seen in MM1/MV1 +2c type	Surgery
76/F	Probable	MV		486	–	+	–	NA		Otolaryngology
66/M	Definite	VV	2	134	+	+	–	NA	Morphological/histochemical pattern as seen in VV2 type	Surgery
74/F	Probable			123	+	+	–	NA		Pediatrics
74/M	Probable				+	+	–	NA		Gynecology
65/M	Definite			809	–	NA	NA	NA	Morphological/histochemical pattern not attributable to a certain disease type	Gynecology
53/M	Definite	VV		175	+	+	+	NA	Morphological/histochemical pattern as seen in VV2 type	Trauma surgery, orthopedic surgery
60/F	Probable	MV		165	+	+	+	–		Surgery
73/M	Definite			49	–	–	–	NA	Deposition of PrP; progressed autolysis, no pattern distinguishable	Internal medicine / nephrology
58/M	Probable			127	+	–	+	+		Orthopedic surgery
83/M	Probable	MM		84	+	+	+	+		Trauma surgery, proctology
75/M	Definite			56	–	+	+	NA	Morphological/histochemical pattern as seen in MM1/MV1 type	General practice
69/M	Probable			110	+	+	+	+		Internal medicine
69/M	Definite	MM	2	93	+	+	+	NA	Morphological/histochemical pattern as seen in MM2 type	Orthopedic surgery
69/M	Probable			100	+	+	–	+		Trauma surgery, orthopedic surgery
61/M	Definite	MM	1	89	+	+	+	+	Morphological/histochemical pattern as seen in MM1/MV1 type	Otolaryngology
70/M	Definite	MM	1	93	+	–	–	+	Morphological/histochemical pattern as seen in MM1/MV1 type	Internal medicine/ general practice

*CJD, Creutzfeldt-Jakob disease; CSF, cerebrospinal fluid; EEG, electroencephalogram; MM, methionine homzygosity; MRI, magnetic resonance imaging; NA, test not performed or no results available; RT-QuIC, real-time quaking induced conversion; VV, valine homozygosity; MV, heterozygosity; +, positive (14–3-3) or CJD-typical results (MRI and EEG); –, negative (14–3-3) or no CJD-typical results (MRI and EEG).
†MRI and EEG were rated according to World Health Organization and its amended criteria by Zerr et al. 2009 (*1*)

Reference

1. Zerr I, Kallenberg K, Summers DM, Romero C, Taratuto A, Heinemann U, et al. Updated clinical diagnostic criteria for sporadic Creutzfeldt-Jakob disease. [Erratum in: Brain. 2012;135] [Pt 4]. Brain. 2009;132:2659–68. PubMed https://doi.org/10.1093/brain/awp191
